# The Repertoire of Glycosphingolipids Recognized by *Vibrio cholerae*

**DOI:** 10.1371/journal.pone.0053999

**Published:** 2013-01-21

**Authors:** John Benktander, Jonas Ångström, Hasse Karlsson, Omid Teymournejad, Sara Lindén, Michael Lebens, Susann Teneberg

**Affiliations:** 1 Institute of Biomedicine, Department of Medical Biochemistry and Cell Biology, University of Gothenburg, Göteborg, Sweden; 2 Institute of Biomedicine, Department of Medical Microbiology and Immunology, University of Gothenburg, Göteborg, Sweden; University of Helsinki, Finland

## Abstract

The binding of cholera toxin to the ganglioside GM1 as the initial step in the process leading to diarrhea is nowadays textbook knowledge. In contrast, the knowledge about the mechanisms for attachment of *Vibrio cholerae* bacterial cells to the intestinal epithelium is limited. In order to clarify this issue, a large number of glycosphingolipid mixtures were screened for binding of El Tor *V. cholerae*. Several specific interactions with minor complex non-acid glycosphingolipids were thereby detected. After isolation of binding-active glycosphingolipids, characterization by mass spectrometry and proton NMR, and comparative binding studies, three distinct glycosphingolipid binding patterns were defined. Firstly, *V. cholerae* bound to complex lacto/neolacto glycosphingolipids with the GlcNAcβ3Galβ4GlcNAc sequence as the minimal binding epitope. Secondly, glycosphingolipids with a terminal Galα3Galα3Gal moiety were recognized, and the third specificity was the binding to lactosylceramide and related compounds. *V. cholerae* binding to lacto/neolacto glycosphingolipids, and to the other classes of binding-active compounds, remained after deletion of the chitin binding protein GbpA. Thus, the binding of *V. cholerae* to chitin and to lacto/neolacto containing glycosphingolipids represents two separate binding specificities.

## Introduction

Diarrheal disease caused by *Vibrio cholerae* remains a major health problem in many parts of the world, leading to 100 000 deaths annually [1]. Infecting *V. cholerae* adhere to the small intestinal epithelium, and cause diarrhea primarily by the production of cholera toxin (CT). CT consists of one A-subunit with enzymatic activity, and five B-subunits mediating binding of the toxin to the small intestinal epithelium. In the early 1970s the GM1 ganglioside was identified as the receptor for CT [2]. Since then a large collection of data about the molecular details of the GM1 binding by CT, and the mechanisms for induction of diarrhea, has accumulated [3].

There are more than 200 O-antigen serogroups of *V. cholerae* known to date, but epidemic cholera is caused only by strains belonging to the O1 and O139 serogroups. The *V. cholerae* O1 strains are divided into two biotypes, designated classical and El Tor, that have evolved from separate lineages [4]. The first six pandemics were caused by the classical biotype, but after 1961 it has been displaced by the El Tor biotype. Recently, an evolution among El Tor strains with emergence of hybrid biotype strains with altered cholera toxin has been observed [5].

In contrast to the advanced information available about the interaction between CT and the GM1 ganglioside, the knowledge about the mechanisms for attachment of *V. cholerae* bacterial cells to the intestinal epithelium is sparse. Several hemagglutinins with roles in adherence have been isolated, but their precise binding specificities have not been defined [4]. Recently, a chitin-binding protein (GbpA), which mediates attachment of *V. cholerae* to chitin surfaces, human intestinal cells and mouse intestine, was characterized [6], [7]. These interactions are inhibited by GlcNAc, but since chitin *per se* is not present on the intestinal epithelium, the carbohydrate sequences required for GbpA-mediated intestinal attachment have not been elucidated.

In the present study we searched for *V. cholerae* carbohydrate recognition by binding of *V. cholerae* to a large number of glycosphingolipid mixtures. Several specific interactions with minor non-acid glycosphingolipids were thereby detected, and after isolation of binding-active glycosphingolipids, and characterization by mass spectrometry and ^1^H NMR, three distinct modes of glycosphingolipid binding were defined.

## Materials and Methods

### *Vibrio cholerae* Strains, Culture Conditions and Labeling

The El Tor *V. cholerae* strain JBK 70 (El Tor Inaba; CTA^−/^CTB^−^) [8], and the classical strain CVD103 (Classical Inaba; CTA^−/^CTB^+^) [9], were first cultured aerobically on Luria agar at 37°C for 12 h. Then colonies were inoculated in AKI medium [10], and incubated at 30°C with shaking under aerobic conditions for 12 h. For metabolic labeling, the medium (10 ml) was supplemented with 10 µl ^35^S-methionine (400 µCi; Amersham Pharmacia Biotech). The bacteria were harvested by centrifugation, washed three times with PBS (phosphate-buffered saline, pH 7.3), and resuspended in PBS containing 2% (w/v) bovine serum albumin, 0.1% (w/v) NaN_3_ and 0.1% (w/v) Tween 20 (BSA/PBS/TWEEN) to a bacterial density of 1×10^8^ CFU/ml. The specific activity of the suspensions was approximately 1 cpm per 100 bacteria.

### Construction of *gbpA* Deletion Mutant Strains

The *gbpA* genes in two different *V. cholerae* strains were disrupted by insertional mutagenesis using a suicide plasmid. One was the El Tor strain JBK70, and the other was the classical strain JS1569, a rifampicin resistant mutant of CVD103 [9].

A 520 base-pair fragment from the central region of the *gbpA* gene was amplified from strain JS1569 using primers gbpAf2 (5′-*GGGGAGATCT*AAACTCGCGTGTTTGATAACGAG-3′) and gbpAr2 (5′-*GGGGAGATCT*TGCAGATTGCCATCGCGATC-3′). The regions of the primers in italics are non-homologous tails that contain a recognition site for the restriction endonuclease XbaI. The underlined nucleotide in primer was changed from C to A in order to introduce a stop codon in the *gbpA* gene in addition to the insertion of suicide plasmid. The amplified DNA was digested with XbaI and ligated into the synthetic R6K-based suicide vector pMT-suicide1 [11]. Ligated DNA was transformed into the *E. coli* strain S17-1 [12], and positive clones were isolated on the basis of restriction analysis of plasmids isolated from chloramphenicol resistant transformants. The resulting plasmid was then transferred into the recipient strains of *V. cholerae* by transconjugation accomplished by patch mating and selection of transconjugants by resistance to chloramphenicol and either rifampicin (JS1569) or polymyxin B (JBK70). The presence of the insert in the chromosomes of the resulting transconjugants was confirmed by PCR using primers gbpAf1 (5′-GCCAACCACGTCACAAAGGATTCC-3′) and gbpAr1 (5′-GAGTGGAGAGGTAGCCACTGGAG-3′) which gave a fragment 2.5 kb larger than the 1.2 kb wild-type due to insertion of the pMT-suicide1 plasmid and a repeat of a 520 bp fragment of the *gbpA* gene. The resulting *V. cholerae* strains were given the strain numbers 1382 (El Tor) and 1375 (classical). Culture and labeling of these *gbpA* deletion mutant strains was done using the same conditions as above, with addition of chloramphenicol 12.5 µg/ml to the agar plates and the AKI medium.

### Pretreatment of Bacterial Cells

Suspensions of radiolabeled *V. cholerae* El Tor were prepared as described above, and suspended in PBS. Prior to incubation on thin-layer chromatograms the bacteria were subjected to one of the following treatments: (I) incubation at 37°C for 60 min; (II) incubation at 65°C for 60 min; (III) treatment with trypsin (Sigma) 1 mg/ml at 37°C for 60 min.

### Chromatogram Binding Assays

Thin-layer chromatography was done on aluminum-backed silica gel 60 high performance thin-layer chromatography plates (Merck, Darmstadt, Germany). Glycosphingolipid mixtures (20–40 µg), or pure glycosphingolipids (1–4 µg), were applied to the plates, and eluted with chloroform/methanol/water (60∶35:8, by volume). Chemical detection was done with anisaldehyde [13].

Binding of radiolabeled bacteria to glycosphingolipids on thin-layer chromatograms was done as described [14]. Dried chromatograms were dipped in diethylether/*n*-hexane (1∶5 v/v) containing 0.5% (w/v) polyisobutylmethacrylate for 1 min. The chromatograms were blocked with BSA/PBS/TWEEN for 2 h at room temperature. Then the plates were incubated for 2 h at room temperature with ^35^S-labeled bacteria (1–5×10^6 ^cpm/ml) diluted in BSA/PBS/TWEEN. After washing six times with PBS, and drying, the plates were autoradiographed for 12–36 h using XAR-5 x-ray films (Eastman Kodak, Rochester, NY). Chromatogram binding assays with ^125^I-labeled lectin from *Erythrina cristagalli* were done as described [15].

### Isolation of El Tor Binding Glycosphingolipids

Total acid and non-acid glycosphingolipid fractions were isolated by standard methods [13]. The non-acid glycosphingolipid fractions were separated by repeated silicic acid chromatography, and final separation was achieved by HPLC or by chromatography on Iatrobeads (Iatrobeads 6RS-8060; Iatron Laboratories, Tokyo) columns, eluted with chloroform/methanol/water 65∶25:4 (by volume), followed by chloroform/methanol/water 60∶35:8 (by volume), and finally chloroform/methanol/water 40∶40:12 (by volume). The fractions obtained were analyzed by thin-layer chromatography and anisaldehyde staining, and the *V. cholerae* El Tor binding activity was assessed using the chromatogram binding assay. The fractions were pooled according to the mobility on thin-layer chromatograms and their El Tor binding activity.

### Endoglycoceramidase Digestion and Liquid Chromatography/Electrospray Ionization Mass Spectrometry

Endoglycoceramidase II from *Rhodococcus* spp. [16] (Takara Bio Europe S.A., Gennevilliers, France) was used for hydrolysis of glycosphingolipids, and the oligosaccharides obtained were analyzed by capillary-liquid chromatography mass spectrometry and tandem mass spectrometry [17]. In brief, the oligosaccharides were separated on a column (200×0.180 mm) packed in-house with 5 µm porous graphite particles (Hypercarb, Thermo Scientific), and eluted with an acetonitrile gradient (A: 10 mM ammonium bicarbonate; B: 100% acetonitrile). The saccharides were analyzed in the negative ion mode on an LTQ linear quadrupole ion trap mass spectrometer (Thermo Electron, San José, CA). The IonMax standard ESI source on the LTQ mass spectrometer was equipped with a stainless steel needle kept at –3.5 kV. Compressed air was used as nebulizer gas. The heated capillary was kept at 270°C, and the capillary voltage was –50 kV. Full-scan (*m/z* 380–2 000, 2 microscans, maximum 100 ms, target value of 30 000) was performed, followed by data dependent MS^2^ scans of the three most abundant ions in each scan (2 microscans, maximum 100 ms, target value of 10 000). The threshold for MS^2^ was set to 500 counts. Normalized collision energy was 35%, and an isolation window of 3 u, an activation q = 0.25, and an activation time of 30 ms, was used. The conditions for MS^3^ and MS^4^ were the same, except that the thresholds were set to 300 and 100 counts, respectively.

### Proton NMR Spectroscopy

^1^H NMR spectra were acquired on a Varian 600 MHz spectrometer at 30°C. Samples were dissolved in dimethyl sulfoxide/D_2_O (98∶2, by volume) after deuterium exchange.

## Results

### Screening for *V. cholerae* Carbohydrate Recognition

**Figure 1:**
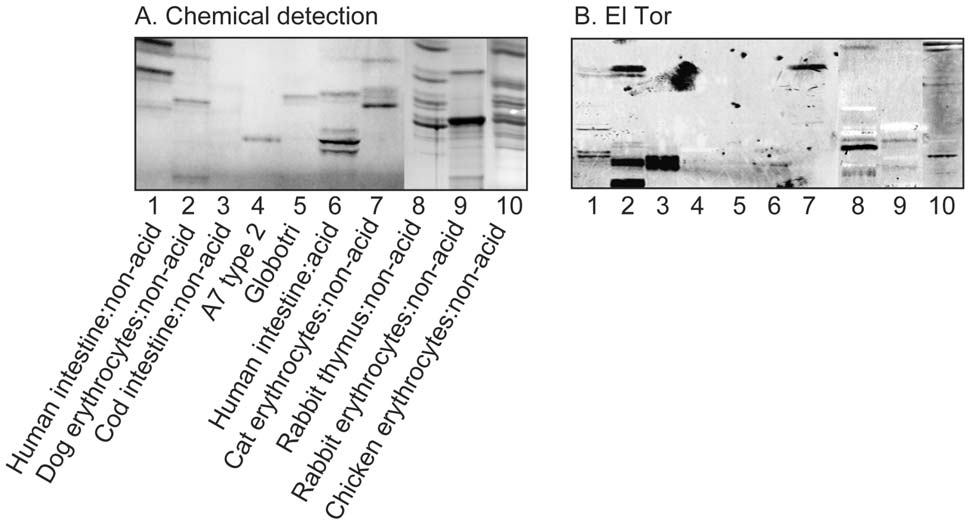
Binding of *V. cholerae* El Tor to mixtures of glycosphingolipids. (A) Chemical detection by anisaldehyde. (B) Autoradiograms obtained by binding of *V. cholerae* JBK 70. The lanes were: Lane 1, non-acid glycosphingolipids of human small intestine, 20 µg; Lane 2, non-acid glycosphingolipids of dog erythrocytes, 20 µg; Lane 3, non-acid glycosphingolipids of cod intestine, 20 µg; Lane 4, A type 2 heptaosylceramide (GalNAcα3(Fucα2)Galβ4(Fucα3)GlcNAcβ3Galβ4Glcβ1Cer), 4 µg; Lane 5, globotriaosylceramide (Galα4Galβ4Glcβ1Cer), 4 µg; Lane 6, acid glycosphingolipids of human small intestine, 40 µg; Lane 7, non-acid glycosphingolipids of cat erythrocytes, 40 µg; Lane 8, non-acid glycosphingolipids of rabbit thymus, 40 µg; Lane 9, non-acid glycosphingolipids of rabbit erythrocytes, 40 µg; Lane 10, non-acid glycosphingolipids of chicken erythrocytes, 40 µg.

In the initial screening for carbohydrate recognition by *V. cholerae*, mixtures of glycosphingolipids from various sources were used in order to expose the bacteria to a large number of potentially binding-active carbohydrate structures. Thus, the binding of the bacteria to acid and non-acid glycosphingolipid mixtures isolated from the small intestine of different species (human, rat, cat, rabbit, dog, monkey and pig), erythrocytes of different species (human, cat, rabbit, dog, horse, chicken and sheep), human cancers (lung cancer, kidney cancer, colon cancer, liver cancer and gastric cancer), and rabbit thymus, was tested. In most non-acid glycosphingolipid fractions a binding of both the El Tor strain and the classical strain to compounds migrating in the mono- and diglycosylceramide regions was observed (exemplified in Figure 1, lanes 2, 7, 8, 10). In addition, a number of slow-migrating minor glycosphingolipids in non-acid fractions of human small intestine, dog erythrocytes, cod intestine, rabbit thymus, rabbit erythrocytes, and chicken erythrocytes were distinctly recognized by the El Tor strain (Figure 1, lanes 1–3, 8–10).

### Characterization of the El Tor Binding Slow-migrating Glycosphingolipid of Human Small Intestine

After separation of the non-acid fraction from human small intestine, the subfractions obtained were pooled according to El Tor binding activity, giving a subfraction containing the slow-migrating El Tor binding compound (designated fraction HI-I).

Based on the observations listed below fraction HI-I was characterized as Galβ3GlcNAcβ4Galβ3(Fucα4)GlcNAcβ3Galβ4Glcβ1Cer, *i.e*. a Le^a^ pentaosylceramide substituted with a terminal lacto moiety.

**Figure 2:**
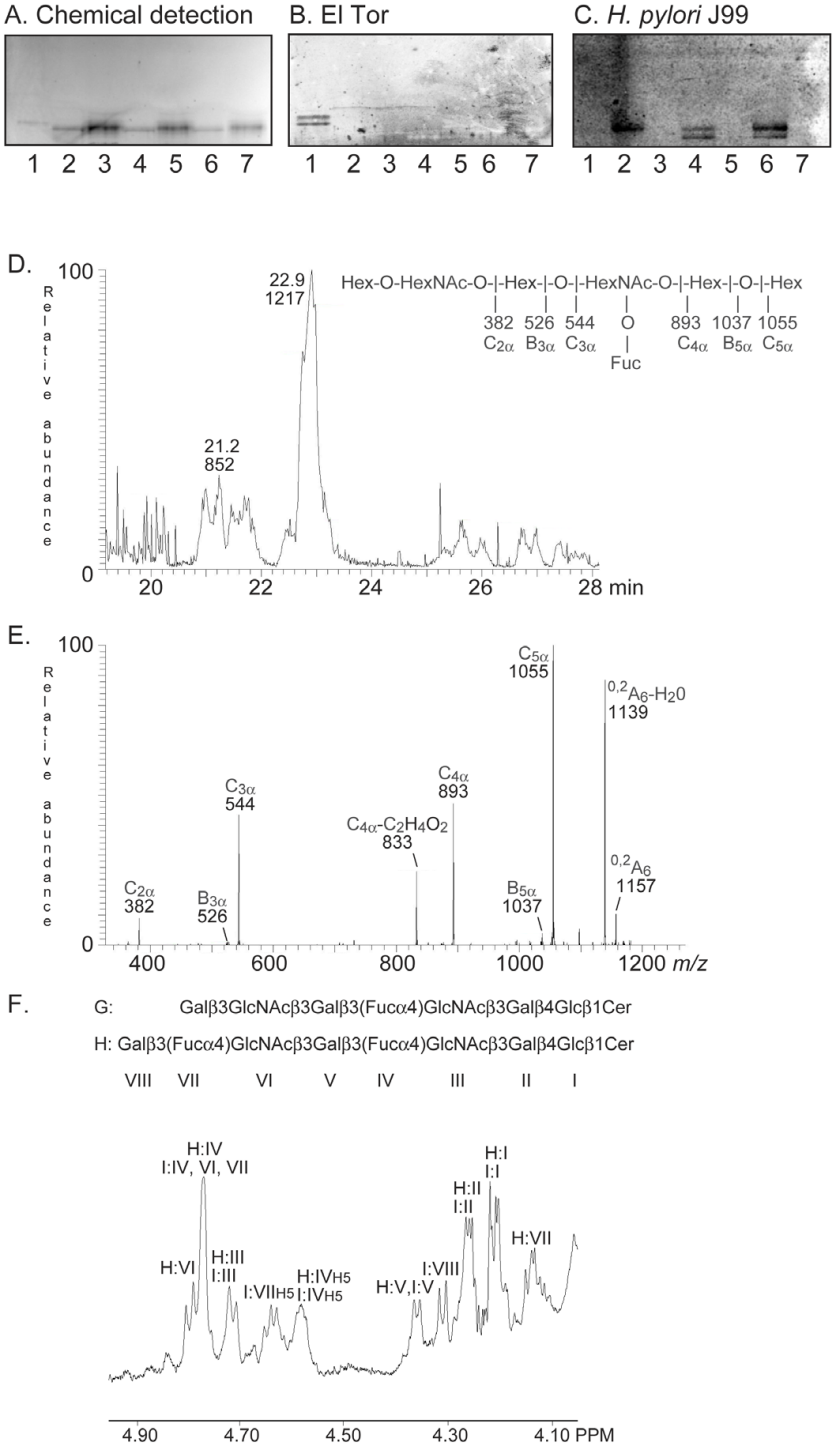
Characterization of the El Tor binding glycosphingolipid of human small intestine. (A) Chemical detection by anisaldehyde. (B) Autoradiogram obtained by binding of *V. cholerae* strain JBK 70. (C) Autoradiogram obtained by binding of *H. pylori* strain J99. The lanes were: Lane 1, fraction HI-I from human small intestine, 4 µg; Lane 2, NeuAcα3Galβ4(Fucα3)GlcNAcβ3Galβ4GlcNAcβ3Galβ4Glcβ1Cer (sialyl-Le^x^-octaosylceramide), 4 µg; Lane 3, Galβ4(Fucα3)GlcNAcβ3Galβ4GlcNAcβ3Galβ4Glcβ1Cer (Le^x^-heptaosylceramide), 4 µg; Lane 4, NeuAcα3Galβ4GlcNAcβ3Galβ4(Fucα3)GlcNAcβ3Galβ4Glcβ1Cer (VIM2), 4 µg; Lane 5, Galβ4GlcNAcβ3Galβ4(Fucα3)GlcNAcβ3Galβ4Glcβ1Cer (de-sialo VIM2), 4 µg; Lane 6, NeuAcα3Galβ4(Fucα3)GlcNAcβ3Galβ4(Fucα3)GlcNAcβ3Galβ4Glcβ1Cer (sialyl-dimeric-Le^x^), 4 µg; Lane 7, Galβ4(Fucα3)GlcNAcβ3Galβ4(Fucα3)GlcNAcβ3Galβ4Glcβ1Cer (dimeric-Le^x^), 4 µg. The sialic acid binding *H. pylori* strain J99 (C) was used as a control for removal of sialic acid from the compounds in lanes 3, 5 and 7 [14]. (D) Base peak chromatogram from LC-ESI/MS of the oligosaccharides derived from fraction HI-I by hydrolysis with *Rhodococcus* endoglycoceramidase II. (E) MS^2^ spectrum of the [M-H^+^]^−^ ion at *m/z* 1217 (retention time 22.9 min). (F) Anomeric region of the 600 MHz ^1^H NMR spectrum of fraction HI-I (30°C). The designations G and H refer to Table 1.

1. On thin-layer chromatograms the El Tor binding glycosphingolipid migrated in the hexa−/heptaglycosylceramide region (Figure 1, lane 1; Figure 2A–B, lane 1).
2. LC-ESI/MS of the oligosaccharides released from fraction HI-I by hydrolysis with endoglycoceramidase II gave two molecular ions (Figure 2D). The major [M-H^+^]^−^ ion at *m/z* 1217 indicated an oligosaccharide with one Fuc, two HexNAc and four Hex, while the minor [M-H^+^]^−^ ion at *m/z* 852 indicated an oligosaccharide with one Fuc, one HexNAc and three Hex. The MS^2^ spectrum of the [M-H^+^]^−^ ion at *m/z* 852 was characteristic for the Le^a^ pentasaccharide [18] (data not shown). MS^2^ of the [M-H^+^]^−^ ion at *m/z* 1217 gave a C-type fragment ion series (C_2α_ at *m/z* 382, C_3α_ at *m/z* 544, C_4α_ at *m/z* 893, and C_5α_ at *m/z* 1055) demonstrating a Hex-HexNAc-Hex-(Fuc)HexNAc-Hex-Hex sequence (Figure 2E). Intense cross-ring ^0,2^A-type fragments are diagnostic for carbohydrates substituted at C-4 [17]–[19]. The ^0,2^A_6_ ion at *m/z* 1157, and the ^0,2^A_6_-H_2_O ion at *m/z* 1139, were derived from cross-ring cleavage of the 4-substituted Glc of the lactose unit at the reducing end. However, no ^0,2^A_2_ fragment ion at *m/z* 281 (not shown), or ^0,2^A_4_ fragment ion at *m/z* 646, were observed, suggesting that the HexNAcs were substituted at 3-position.
3. ^1^H NMR of fraction HI-I shows a mixture of three compounds, all with Le^a^ signatures (Figure 2F). The two major structures (∼35% and 60%, respectively) are Galβ3(Fucα4)GlcNAcβ4Galβ3(Fucα4)GlcNAcβ3Galβ4Glcβ1Cer previously characterized by NMR [20], and its precursor Galβ3GlcNAcβ4Galβ3(Fucα4)GlcNAcβ3Galβ4Glcβ1Cer in which the terminal Fucα4 is lacking resulting in a lacto terminal, as found above by mass spectrometry. This structural difference results in drastic shifts of the anomeric resonances arising from the lacto terminal relative to the same residues in the Le^a^ terminal: downfield by +0.034 ppm for GlcNAcβ3 and upfield for Galβ3 by −0.169 ppm, thus yielding shifts corresponding to those seen for the lacto-terminated hexaosylceramide at 4.794 ppm and 4.14 ppm (Table 1). All other resonances of the lacto-terminated compound overlap completely with those of the dimeric Le^a^ structure (Figure 2F; Table 1). The third minor compound (∼5%) is the Le^a^ pentaosylceramide (Galβ3(Fucα4)GlcNAcβ3Galβ4Glcβ1Cer).

**Table 1:** Chemical shift data (ppm) for anomeric resonances from proton NMR spectra at 600 MHz of *V. cholerae* binding glycosphingolipids, and co-migrating glycosphingolipids, obtained in DMSO-d_6_/D_2_O (98∶2, by volume) at 30°C.

	X	IX	VIII	VII	VI	V	IV	III	II	I		Fraction
**A**						Galα3	Galβ4	GlcNAcβ3	Galβ4	Glcβ1	Cer	TH-II, RE-I
						4.826	4.279	4.627	4.25	4.16		
**B**						GlcNAcβ3	Galβ4	GlcNAcβ3	Galβ4	Glcβ1	Cer	TH-I
						4.604	4.250	4.640	4.250	4.15		
**C**					Galβ4	GlcNAcβ3	Galβ4	GlcNAcβ3	Galβ4	Glcβ1	Cer	TH-II
					4.20	5.643	4.253	4.643	4.253	4.16		
**D**					Galα3	Galα3	Galβ4	GlcNAcβ3	Galβ4	Glcβ1	Cer	RE-I
					4.826	4.893	4.28	4.66	4.25	4.16		
**E**					Galβ4	GlcNAcβ3	Galβ4	GlcNAcβ3	Galβ4	Glcβ1	Cer	TH-III
					4.13	4.796	4.25	4.64	4.25	4.16		
**F**				Galα3	Galβ4	GlcNAcβ3	Galβ4	GlcNAcβ3	Galβ4	Glcβ1	Cer	TH-III, IV
				4.826	4.279	4.626	4.257	4.78	4.257	4.16		
**G**						Galβ3	(Fucα4)	GlcNAcβ3	Galβ4	Glcβ1	Cer	HI-I
						4.293	4.767	4.727	4.260	4.207		
**H**				Galβ3	GlcNAcβ3	Galβ3	(Fucα4)	GlcNAcβ3	Galβ4	Glcβ1	Cer	HI-I
				4.14	4.794	4.358	4.77	4.712	4.26	4.21		
**I**			Galβ3	(Fucα4)	GlcNAcβ3	Galβ3	(Fucα4)	GlcNAcβ3	Galβ4	Glcβ1	Cer	HI-I
			4.309	4.77	4.76	4.358	4.77	4.712	4.26	4.21		
**J**			Galβ4	GlcNAcβ3	Galβ4	GlcNAcβ3	Galβ4	GlcNAcβ3	Galβ4	Glcβ1	Cer	TH-IV
			4.209	4.651	4.255	4.651	4.255	4.651	4.255	4.16		
**K**	Galα3	Galβ4	GlcNAcβ6	(Galα3	Galβ4	GlcNAcβ3)	Galβ4	GlcNAcβ3	Galβ4	Glcβ1	Cer	DE-I
	4.827	4.282	4.395	4.827	4.282	4.652	4.290	4.652	4.254	4.161		

### Characterization of the El Tor Binding Slow-migrating Glycosphingolipids of Rabbit Thymus

After separation of the non-acid glycosphingolipid fraction from rabbit thymus, and pooling of the subfractions obtained according El Tor binding activity, four El Tor binding fractions were obtained, designated fraction TH-I, TH-II, TH-III, and TH-IV, respectively. These fractions were characterized by mass spectrometry, ^1^H NMR, and binding of the Galβ4GlcNAcβ-binding *E. cristagalli* lectin [14], as follows.

#### A. Fraction TH-I

**Figure 3:**
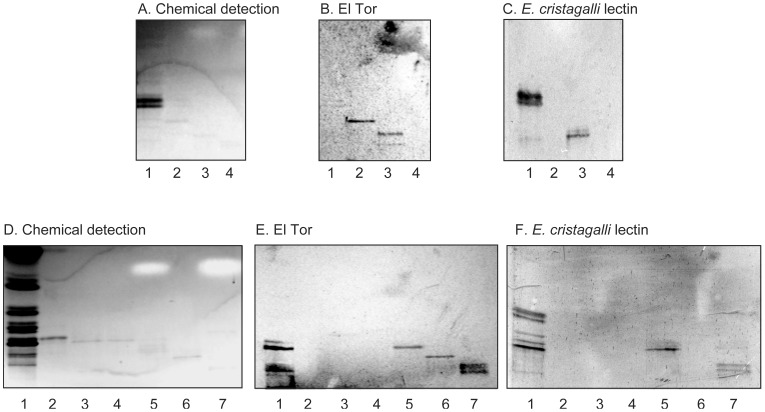
Binding of *V. cholerae* El Tor and *E. cristagalli* lectin to glycosphingolipids of rabbit thymus. (A and D) Chemical detection by anisaldehyde. (B and E) Autoradiograms obtained by binding of *V. cholerae* JBK 70. (C and F) Autoradiograms obtained by binding of *E. cristagalli* lectin. The lanes on A–C were: Lane 1, neolactotetraosylceramide (Galβ4GlcNAcβ3Galβ4Glcβ1Cer), 4 µg; Lane 2, fraction TH-I isolated from rabbit thymus, 1 µg; Lane 3, fraction TH-II from rabbit thymus, 1 µg; Lane 4, Lane 4, sialylneolactohexaosylceramide (NeuGcα3Galβ4GlcNAcβ3Galβ4GlcNAcβ3Galβ4Glcβ1Cer), 1 µg. The lanes on D–F were: Lane 1, total non-acid glycosphingolipids of rabbit thymus, 40 µg; B5 pentaosylceramide (Galα3Galβ4GlcNAcβ3Galβ4Glcβ1Cer), 4 µg; Lanes 3 and 4, subfractions isolated from rabbit thymus, 1 µg/lane; Lane 5, fraction TH-II from rabbit thymus, 1 µg; Lane 6, fraction TH-III, 1 µg; Lane 7, fraction TH-IV, 1 µg.

**Figure 4:**
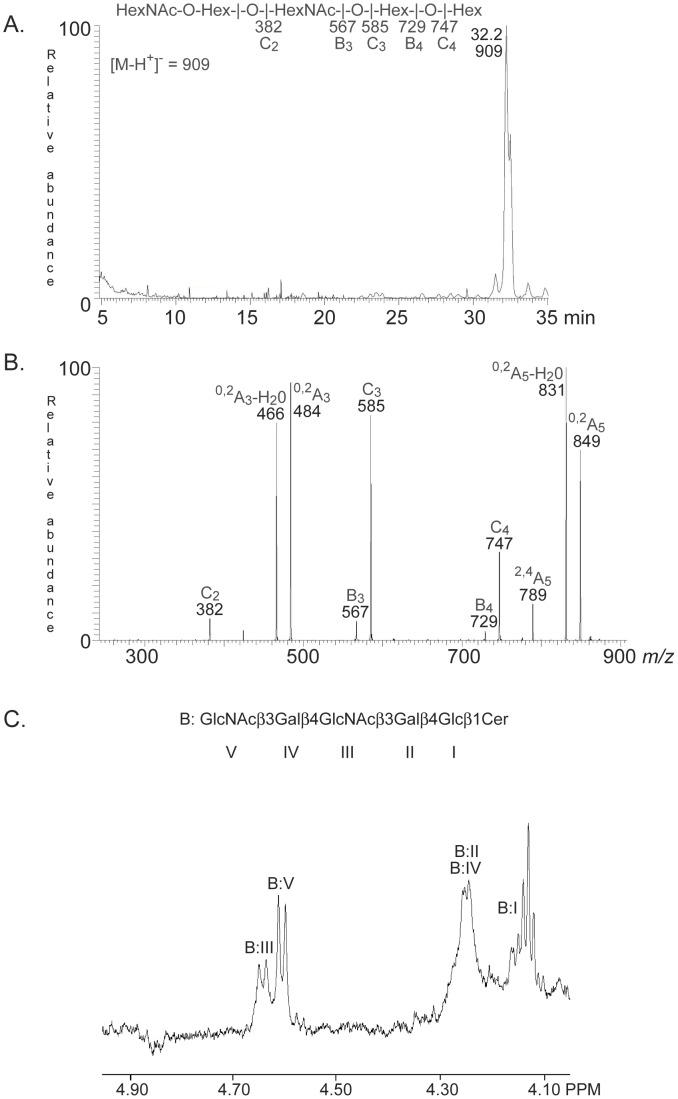
Characterization of El Tor binding fraction TH-I from rabbit thymus. (A) Base peak chromatogram from LC-ESI/MS of the oligosaccharide obtained by digestion of fraction TH-I with endoglycoceramidase II. (B) MS^2^ spectrum of the [M-H^+^]^−^ ion at *m/z* 909 (retention time 32.2 min). (C) Anomeric region of the 600 MHz ^1^H NMR spectrum of fraction TH-I (30°C). The designation B refer to Table 1.

Fraction TH-I was characterized as GlcNAcβ3Galβ4GlcNAcβ3Galβ4Glcβ1Cer. This conclusion is based on the following observations:

1. On thin-layer chromatograms fraction TH-I migrated in the pentaglycosylceramide region (Figure 3B, lane 2).
2. No binding of the *E. cristagalli* lectin to fraction TH-I was obtained (Figure 3C, lane 2).
3. LC-ESI/MS of the oligosaccharides obtained by hydrolysis of fraction TH-1 gave a major [M-H^+^]^−^ ion at *m/z* 909, demonstrating an oligosaccharide with two HexNAc and three Hex (Figure 4A). MS^2^ of the ion at *m/z* 909 resulted in a series of C-type fragment ions (C_2_ at *m/z* 382, C_3_ at *m/z* 585, and C_4_ at *m/z* 747) identifying a pentasaccharide with HexNAc-Hex-HexNAc-Hex-Hex sequence (Figure 4B). The ^0,2^A_3_ fragment ion at *m/z* 484, and the ^0,2^A_3_-H_2_O fragment ion at *m/z* 466, indicated a 4-substituted internal HexNAc. The ^0,2^A_5_ ion at *m/z* 849, and the ^0,2^A_5_-H_2_O ion at *m/z* 831, were derived from cross-ring cleavage of the 4-substituted Glc of the lactose part.
4. The proton NMR spectrum of fraction TH-1 in Figure 4C reveals a single species showing two GlcNAcβ3 residues at 4.603 ppm and 4.641 ppm, two overlapping Galβ4 residues at 4.25 ppm and a Glcβ1 at 4.15 ppm, consistent with the structure GlcNAcβ3Galβ4GlcNAcβ3Galβ4Glcβ1Cer [21] (Table 1).

#### B. Fraction TH-II

Characterization of the El Tor binding fraction TH-II demonstrated neolactohexaosylceramide (Galβ4GlcNAcβ3Galβ4GlcNAcβ3Galβ4Glcβ1Cer) as the major compound. This conclusion was based on the following properties:

1. On thin-layer chromatograms fraction TH-II migrated in the hexaglycosylceramide region (Figure 3A–B, lane 3; Figure 3D–E, lane 5).
2. Fraction TH-II was recognized by the *E. cristagalli* lectin (Figure 3C, lane 3; Figure 3F, lane 5).
3. LC-ESI/MS of the oligosaccharides obtained by hydrolysis of fraction TH-II gave a major [M-H^+^]^−^ ion at *m/z* 1071, demonstrating an oligosaccharide with two HexNAc and four Hex, and a minor [M-H^+^]^−^ ion at *m/z* 868, indicating an oligosaccharide with one HexNAc and four Hex (data not shown). MS^2^ of the major [M-H^+^]^−^ ion at *m/z* 1071 gave a series of prominent C-type fragment ions (C_2_ at *m/z* 382, C_3_ at *m/z* 544, C_4_ at *m/z* 747, and C_5_ at *m/z* 909) identifying a hexasaccharide with Hex-HexNAc-Hex-HexNAc-Hex-Hex sequence (Figure S1A). A 4-substitution of the internal HexNAc was identified by the ^0,2^A_3_ fragment ion at *m/z* 646, and the ^0,2^A_3_-H_2_O fragment ion at *m/z* 628.
4. The ^1^H NMR spectrum of fraction TH-II (not shown) reveals a mixture of neolactohexaosylceramide, as evidenced by two overlapping GlcNAcβ3 resonances at 5.643 ppm and two overlapping Galβ4 resonances at 4,25 ppm, as well as a third one at 4.20 ppm, and a Glcβ1 resonance at 4.16 ppm yielding Galβ4GlcNAcβ3Galβ4GlcNAcβ3Galβ4Glcβ1Cer [21], and the B5 pentaosylceramide, as seen by resonances at 4.826 ppm (Galα3), 4.627 ppm (GlcNAcβ3) and 4.279 ppm (penultimate Galβ4), which together with the lactosylceramide part yields Galα3Galβ4GlcNAcβ3Galβ4Glcβ1Cer [22] (Table 1).

#### C. Fraction TH-III

The main compound of fraction TH-III was a neolactoheptaosylceramide with a terminal Galili epitope (Galα3Galβ4GlcNAcβ3Galβ4GlcNAcβ3Galβ4Glcβ1Cer). This conclusion was based on the following observations:

1. Fraction TH-III migrated in the heptaglycosylceramide region on thin-layer chromatograms (Figure 3D–E, lane 6).
2. Fraction TH-III was not recognized the *E. cristagalli* lectin (Figure 3F, lane 6).
3. LC-ESI/MS of the oligosaccharides released from fraction TH-III gave a major [M-H^+^]^−^ ion at *m/z* 1233, demonstrating an oligosaccharide with two HexNAc and five Hex (data not shown). A heptasaccharide with Hex-Hex-HexNAc-Hex-HexNAc-Hex-Hex sequence was identified by the series of C-type fragment ions (C_3_ at *m/z* 544, C_4_ at *m/z* 747, C_5_ at *m/z* 909, and C_5_ at *m/z* 1071) obtained by MS^2^ of the ion at *m/z* 1233 (Figure S1B). A terminal Hex-Hex-HexNAc sequence with a substitution at C-4 of the HexNAc was identified by the ^0,2^A_3_ fragment ion at *m/z* 443, and the ^0,2^A_3_-H_2_O fragment ion at *m/z* 425. In the same manner, the ^0,2^A_5_ fragment ion at *m/z* 808, and the ^0,2^A_5_-H_2_O fragment ion at *m/z* 790, indicated a 4-substitution of the internal HexNAc, while the ^0,2^A_7_ ion at *m/z* 1173, and the ^0,2^A_7_-H_2_O ion at *m/z* 1155, were obtained by cross-ring cleavage of the 4-substituted Glc of the lactose unit at the reducing end.
4. The ^1^H NMR spectrum of fraction TH-III (not shown) shows a structure having a Galili terminus elongated by an internal Galβ4GlcNAcβ3 yielding Galα3Galβ4GlcNAcβ3Galβ4GlcNAcβ3Galβ4Glcβ1Cer, having almost identical shift values as the B5 pentaosylceramide (Table 1). A minor second species is also present, seen by a GlcNAcβ3 doublet at 4.794 ppm and a Galβ3 doublet at 4.13 ppm, which indicate a lacto-terminated hexaglycosylceramide Galβ3GlcNAcβ3Galβ4GlcNAcβ3Galβ4Glcβ1Cer [20], where the remaining anomeric signals strongly overlap with those of the other structure (Table 1).

#### D. Fraction TH-IV

El Tor binding fraction TH-IV was characterized as neolactooctaosylceramide (Galβ4GlcNAcβ3Galβ4GlcNAcβ3Galβ4GlcNAcβ3Galβ4Glcβ1Cer). This conclusion was based on the following properties:

1. On thin-layer chromatograms fraction TH-IV migrated in the octaglycosylceramide region (Figure 3D–E, lane 7).
2. The *E. cristagalli* lectin bound to fraction TH-IV (Figure 3F, lane 7).
3. LC-ESI/MS of the oligosaccharides released from fraction TH-IV gave a minor [M-H^+^]^−^ ion at *m/z* 1233 as above, and a major [M-H^+^]^−^ ion at *m/z* 1436, demonstrating an oligosaccharide with three HexNAc and five Hex (data not shown). MS^2^ of the ion at *m/z* 1436 gave a series of C-type fragment ions (C_3_ at *m/z* 544, C_4_ at *m/z* 747, C_5_ at *m/z* 909, C_6_ at *m/z* 1112, and C_7_ at *m/z* 1274) indicating an octasaccharide with Hex-HexNAc-Hex-HexNAc-Hex-HexNAc-Hex-HexNAc-Hex-Hex sequence (Figure S1C). As above, the ^0,2^A cross-ring cleavage ions (^0,2^A_4_ at *m/z* 646 and ^0,2^A_6_ at *m/z* 1011) identified substitutions at C-4 of the internal HexNAcs, while the ^0,2^A_8_ ion at *m/z* 1376 was obtained by cross-ring cleavage of the 4-substituted Glc of the lactose unit.
4. The ^1^H NMR spectrum of TH-IV (Figure S1D) is very similar to TH-II revealing a neolactoocta compound and the seven-sugar Galili-terminated structure described above. The chemical shifts are thus identical to those described for fraction TH-II (Table 1).

Thus, the slow-migrating El Tor binding glycosphingolipids of rabbit thymus were characterized as GlcNAcβ3-neolactotetraosylceramide, neolactohexaosylceramide, the B7 heptaosylceramide and neolactooctaosylceramide. In fraction TH-II, the B5 pentaosylceramide was also present, but this compound was not recognized by of *V. cholerae* (see below).

The El Tor binding glycosphingolipids of cod intestine (Figure 1, lane 3) and chicken erythrocytes (Figure 1, lane 10) were also identified as neolactohexaosylceramide by the same set of methods (data not shown). Furthermore, the uppermost El Tor binding glycosphingolipid of dog erythrocytes (Figure 1, lane 2) was characterized as Galα3Galβ4GlcNAcβ6(Galα3Galβ4GlcNAcβ3)Galβ4GlcNAcβ3Galβ4Glcβ1Cer (Figure S2), *i.e*. a branched neolactodecaglycosylceramide with terminal Galili epitopes.

**Figure 5:**
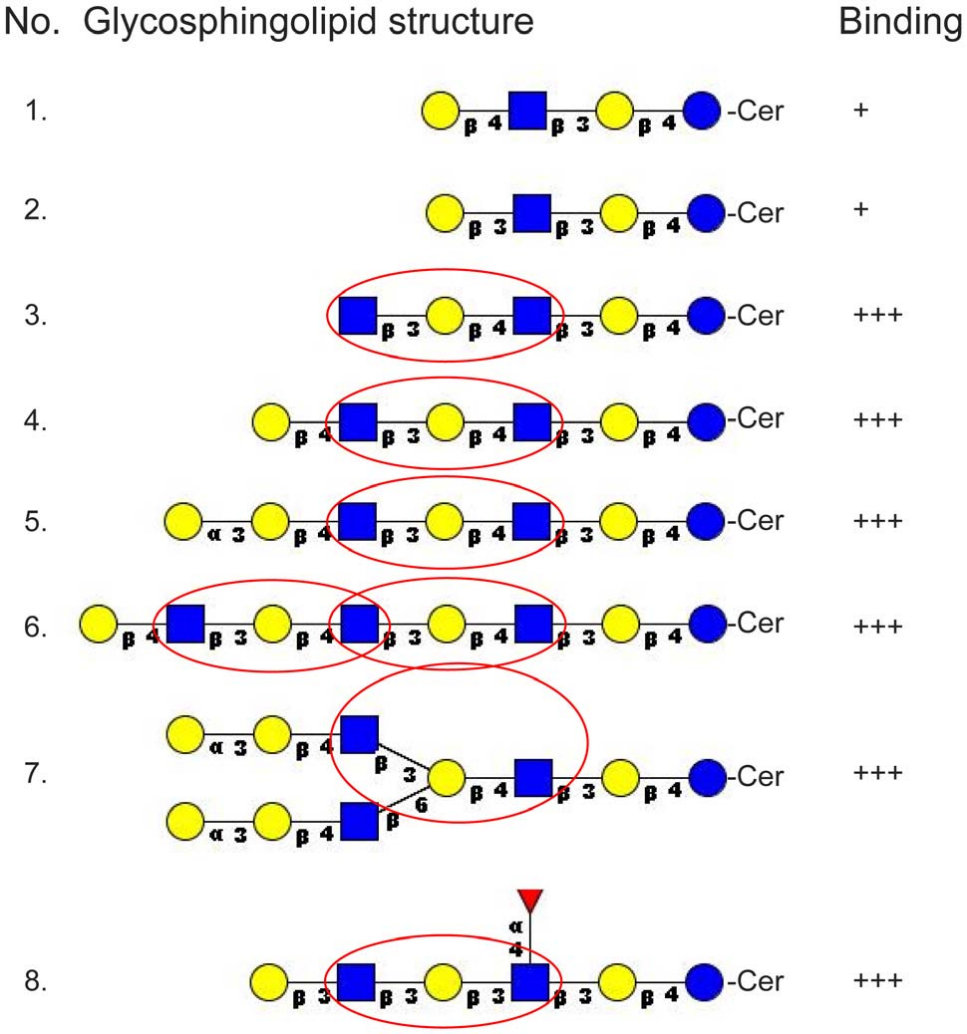
Summary of *Vibrio cholerae* El Tor binding lacto and neolacto glycosphingolipids. The red circles marks the GlcNAcβ3Galβ3/4GlcNAc sequence required for high affinity binding. Blue ring, glucose; yellow ring, galactose; blue square, *N*-acetylglucosamine; red triangle, fucose. Binding is defined as follows:+++denotes a binding when 1 µg of the glycosphingolipid was applied on the thin-layer chromatogram, while+denotes an occasional binding at 4 µg.

Thus, lacto or neolacto sequences were present in all complex El Tor binding glycosphingolipids hitherto characterized. In addition, neolactotetraosylceramide (Galβ4GlcNAcβ3Galβ4Glcβ1Cer) and lactotetraosylceramide (Galβ3GlcNAcβ3Galβ4Glcβ1Cer) were occasionally recognized. Binding to neolactotetraosylceramide and lactotetraosylceramide required 4 µg of the compound on the thin-layer plate, and was only found in approximately 70% of the experiments, indicating that the binding epitope was not optimally exposed in these relatively short glycosphingolipids. For neolactotetraosylceramide an increased binding is obtained by the addition of a terminal GlcNAcβ3 (No. 2), and inspection of the El Tor binding compounds in Figure 5 reveals that the GlcNAcβ3Galβ3/4GlcNAc sequence is the common denominator of all the El Tor binding lacto/neolacto based glycosphingolipids with more than four carbohydrate units.

### Characterization of a Novel Glycosphingolipid of Rabbit Erythrocytes Recognized by *V. cholerae*

**Figure 6:**
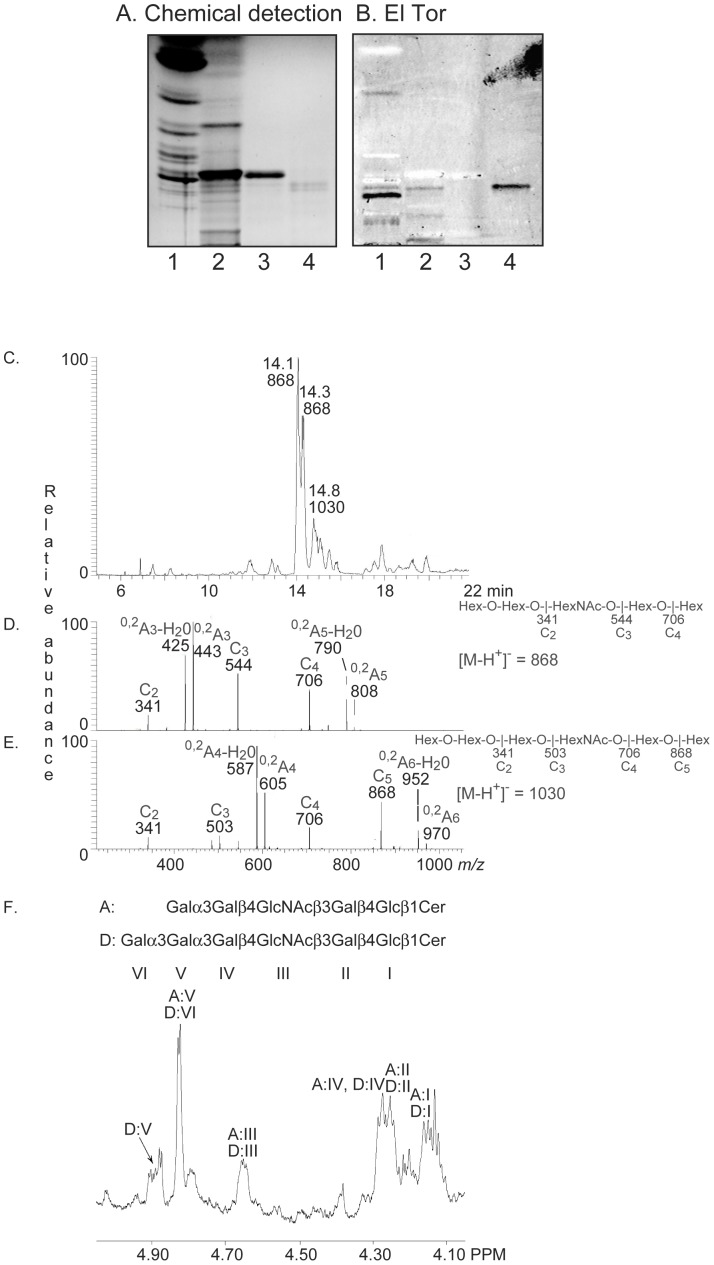
Characterization of the El Tor binding glycosphingolipid of rabbit erythrocytes. (A) Chemical detection by anisaldehyde. (B) Autoradiogram obtained by binding of *V. cholerae* strain JBK 70. The lanes were: Lane 1, total non-acid glycosphingolipids of rabbit thymus, 40 µg; Lane 1, total non-acid glycosphingolipids of rabbit erythrocytes, 40 µg; Lane 3, B5 pentaosylceramide (Galα3Galβ4GlcNAcβ3Galβ4Glcβ1Cer), 4 µg; Lane 4, fraction RE-I of rabbit erythrocytes, 2 µg. (C) Base peak chromatogram from LC-ESI/MS of the oligosaccharides derived from fraction RE-I by hydrolysis with endoglycoceramidase II. (D) MS^2^ spectrum of the [M-H^+^]^−^ ion at *m/z* 868 (retention time 14.0 min). (E) MS^2^ spectrum of the [M-H^+^]^−^ ion at *m/z* 1030 (retention time 14.7 min). (F) Anomeric region of the 600 MHz ^1^H NMR spectrum of fraction RE-I (30^o^C). The designations A and D refer to Table 1.

The non-acid glycosphingolipids of rabbit erythrocytes has been characterized in detail by mass spectrometry and NMR, demonstrating a number of Galα3Galβ4GlcNAc-terminated linear and di−/triantennary oligo- and polyglycosylceramides [22]–[25]. The major non-acid glycosphingolipid of rabbit erythrocytes is the B5 pentaosylceramide, and the El Tor binding glycosphingolipid was found just below this compound (Figure 1B, lane 9; Figure 6B, lane 4). Separation of a non-acid glycosphingolipid fraction from rabbit erythrocytes and pooling of the subfractions obtained according to El Tor binding activity gave a fraction containing the El Tor binding compound (designated fraction RE-I). Structural characterization by mass spectrometry and proton NMR, as detailed below, identified two glycosphingolipids in fraction RE-I; the B5 pentaosylceramide and a novel glycosphingolipid structure, Galα3Galα3Galβ4GlcNAcβ3Galβ4Glcβ1Cer, *i.e* a B5 pentaglycosylceramide elongated by Galα3.

1. LC-ESI/MS of the oligosaccharides obtained by hydrolysis of fraction RE-1 gave a major [M-H^+^]^−^ ion at *m/z* 868, demonstrating an oligosaccharide with one HexNAc and four Hex, and a [M-H]^−^ ion at *m/z* 1030, indicating an oligosaccharide with one HexNAc and four Hex (Figure 6C).

The MS^2^ spectrum of the [M-H^+^]^−^ ion at *m/z* 868 had a C-type fragment ion series (C_2_ at *m/z* 341, C_3_ at *m/z* 544, and C_4_ at *m/z* 706), identifying a Hex-Hex-HexNAc-Hex-Hex sequence (Figure 6D). The ^0.2^A_3_ fragment ion at *m/z* 443, and the ^0,2^A_3_-H_2_O fragment ion at *m/z* 425, demonstrated a 4-substitution of the HexNAc, *i.e*. a type 2 core chain, while the ^0.2^A_5_ fragment ion at *m/z* 808, and the ^0,2^A_5_-H_2_O fragment ion at *m/z* 790, were obtained by cross-ring cleavage of the 4-substituted Glc of the lactose unit.

MS^2^ of the [M-H^+^]^−^ ion at *m/z* 1030 (Figure 6E) gave a series of C-type fragment ions (C_2_ at *m/z* 341, C_3_ at *m/z* 503, C_4_ at *m/z* 706, and C_5_ at *m/z* 868) indicating a Hex-Hex-Hex-HexNAc-Hex-Hex sequence. A type 2 core chain was demonstrated by the ^0,2^A_4_ fragment ion at *m/z* 605, and the ^0,2^A_4_-H_2_O fragment ion at *m/z* 587, which are diagnostic for substitution of the HexNAc at C4.

1. ^1^H NMR of fraction RE-1 reveals a mixture of one major component and several minor ones. The main structure was identified as the B5 pentaosylceramide (Galα3Galβ4GlcNAcβ3Galβ4Glcβ1Cer) from signals at 4.826 ppm (Galα3), 4.65 ppm (GlcNAcβ3), 4.279 ppm (penultimate Galβ4), 4.25 ppm (Galβ4) and 4.16 ppm (Glcβ1) (Figure 6F). The spectrum also had three α-signals clustered around 4.90 ppm, one of which coincides precisely with an internal Galα3 (4.893 ppm) substituted with a terminal Galα3, as found earlier for Galα3Galα3Galβ4Glcβ1Cer [26]. Thus, a structure with the sequence Galα3Galα3Galβ4GlcNAcβ3Galβ4Glcβ1Cer is present, in accord with the mass spectrometry data above, and where the shifts for the remaining sugar residues are very close to those found for the B5 pentaosylceramide (Table 1). The two α-signals seen on either side of the internal Galα3 resonance just mentioned could not be assigned.

### Binding to Reference Glycosphingolipids

**Figure 7:**
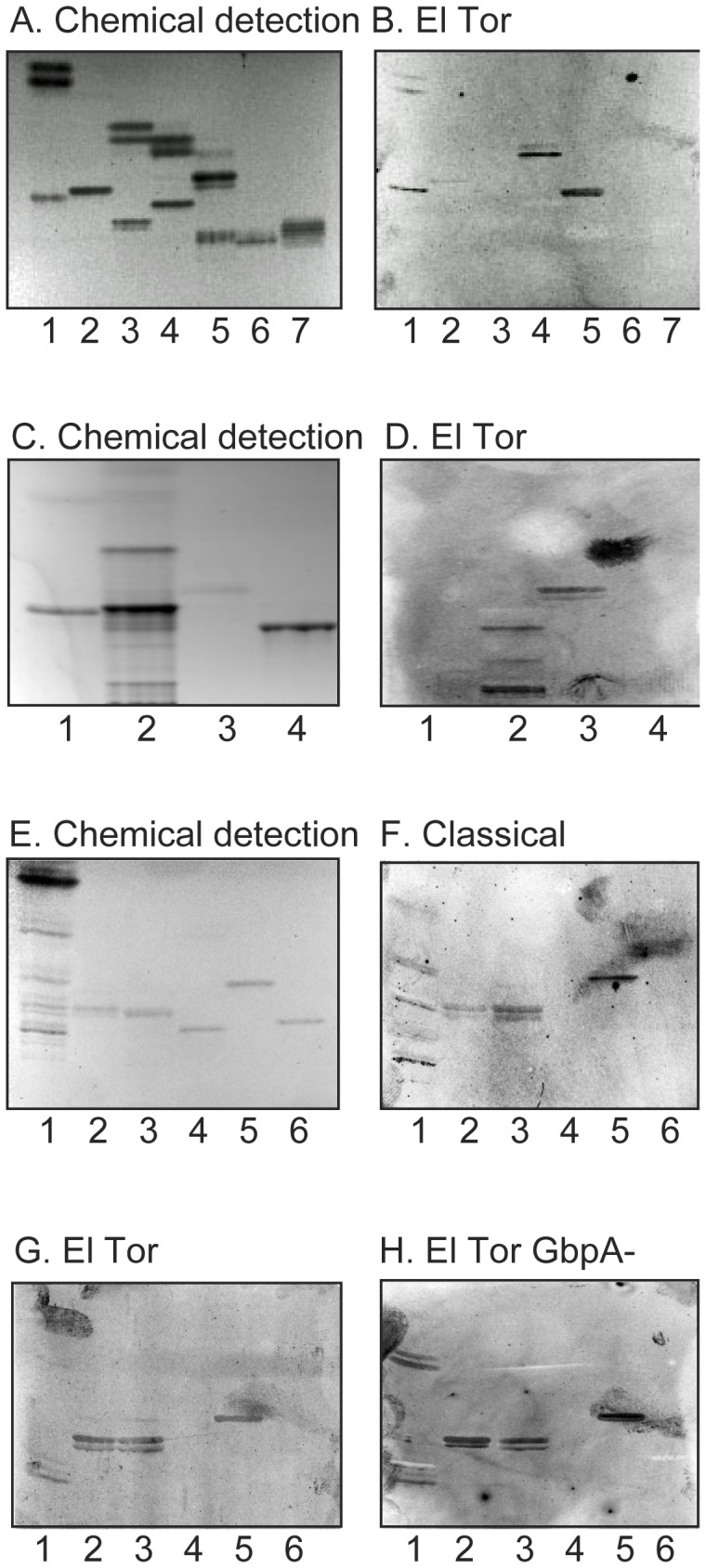
Comparison of the glycosphingolipid binding of classical and El Tor *V. cholerae,* and an El Tor GbpA deletion mutant strain. (A, C, E) Chemical detection by anisaldehyde. (B, D, G) Autoradiograms obtained by binding of El Tor *V. cholerae* strain JBK 70. (F) Autoradiogram obtained by binding of classical *V. cholerae* strain CVD103. (H) Autoradiogram obtained by binding of El Tor GbpA deletion mutant strain 1382. The lanes on (A and B) were: Lane 1, galactosylceramide (Galβ1Cer), 4 µg, and gangliotriaosylceramide (GalNAcβ4Galβ4Glcβ1Cer), 4 µg; Lane 2, glucosylceramide (Glcβ1Cer), 4 µg, and globotriaosylceramide (Galα4Galββ4Glcβ1Cer), 4 µg; Lane 3, lactosylceramide (Galβ4Glcβ1Cer) with sphingosine and non-hydroxy fatty acids, 4 µg, and Forssman pentaosylceramide (GalNAcα3GalNAcβ3Galα4Galβ4Glcβ1Cer), 4 µg; Lane 4, lactosylceramide (Galβ4Glcβ1Cer) with phytosphingosine and hydroxy fatty acids, 4 µg, and globotetraosylceramide (GalNAcβ3Galα4Galβ4Glcβ1Cer), 4 µg; Lane 5, isoglobotriaosylceramide (Galα3Galβ4Glcβ1Cer), 4 µg, and Le^a^ pentaosylceramide (Galβ3(Fucα4)GlcNAcβ3Galβ4Glcβ1Cer), 4 µg; Lane 6, Le^x^ pentaosylceramide (Galβ4(Fucα3)GlcNAcβ3Galβ4Glcβ1Cer), 4 µg; lane 7, H type 1 pentaosylceramide (Fucα2Galβ3GlcNAcβ3Galβ4Glcβ1Cer), 4 µg. The lanes on (C and D) were: Lane 1, B5 pentaosylceramide (Galα3Galβ4GlcNAcβ3Galβ4Glcβ1Cer), 4 µg; Lane 2, non-acid glycosphingolipids of rabbit erythrocytes, 40 µg; Lane 3, Galα3Galα3Galβ4Glcβ1Cer, 1 µg; Lane 4, B type 2 hexaosylceramide (Galα3(Fucα2)Galβ4GlcNAcβ3Galβ4Glcβ1Cer), 4 µg. The lanes on (E-H) were: Lane 1, non-acid glycosphingolipids of rabbit thymus, 20 µg; Lane 2, lactotetraosylceramide (Galβ3GlcNAcβ3Galβ4Glcβ1Cer), 2 µg; Lane 3, Galα3Galα3Galβ4Glcβ1Cer, 4 µg; Lane 4, B5 pentaosylceramide, 4 µg; Lane 5, gangliotriaosylceramide, 4 µg; Lane 6, H type 1 pentaosylceramide, 4 µg.

Finally, the binding of the El Tor strain to a number of reference glycosphingolipids was evaluated (summarized in Table 2). Like many other bacteria, the El Tor strain bound to lactosylceramide with phytosphingosine and hydroxy fatty acids (No. 32 in Table 2; Figure 7B, lane 4, upper band), isoglobotriaosylceramide (No. 33; Figure 7B, lane 5, upper band), galactosylceramide (No. 31; Figure 7B, lane 1, upper band), gangliotriaosylceramide (No. 35; Figure 7B, lane 1, lower band), and gangliotetraosylceramide (No. 36). However, the majority of the tested compounds, as globoseries glycosphingolipids (Nos. 42-44 in Table 2), blood group A/B/H-terminated glycosphingolipids (Nos. 7, 11, 12, 14, 23, 26 and 28), Le^x^-, Le^a^- and Le^b^-terminated glycosphingolipids (Nos. 8, 16, 17, 27, 29 and 30), and the gangliosides (Nos. 18–23 and 46–49), were not recognized by the bacteria.

**Table 2:** Summary of results from binding of *Vibrio cholerae* El Tor to glycosphingolipids on thin-layer chromatograms.

No.	Trivial name	Structure	Source
**I.** **Neolacto binding** ^a^			
**A.** **Binding-active glycosphingolipids**			
1.	*Neolactotetra*	*Galβ4GlcNAcβ3Galβ4Glcβ1Cer*	Human granulocytes
2.	GlcNAcβ3-neolactotetra	**GlcNAcβ3Galβ4GlcNAc**β3Galβ4Glcβ1Cer^b^	Rabbit thymus
3.	Neolactohexa	Galβ4**GlcNAcβ3Galβ4GlcNAc**β3Galβ4Glcβ1Cer	Rabbit thymus
4.	B7	Galα3Galβ4**GlcNAcβ3Galβ4GlcNAc**β3Galβ4Glcβ1Cer	Rabbit thymus
5.	Neolactoocta	Galβ4**GlcNAcβ3Galβ4GlcNAc**β3Galβ4GlcNAcβ3Galβ4Glcβ1Cer	Rabbit thymus
6.	B10	Galα3Galβ4GlcNAcβ6(Galα3Galβ4**GlcNAcβ3**)**Galβ4GlcNAc**β3Galβ4Glcβ1Cer	Dog erythrocytes
**B.** **Related non-binding glycosphingolipids**			
7.	H5-2	Fucα2Galβ4GlcNAcβ3Galβ4Glcβ1Cer	Human erythrocytes
8.	Le^x^-5	Galβ4(Fucα3)GlcNAcβ3Galβ4Glcβ1Cer	Dog intestine
9.	B5	Galα3Galβ4GlcNAcβ3Galβ4Glcβ1Cer	Rabbit erythrocytes
10.	P1	Galα4Galβ4GlcNAcβ3Galβ4Glcβ1Cer	Human erythrocytes
11.	B6-2	Galα3(Fucα2)Galβ4GlcNAcβ3Galβ4Glcβ1Cer	Human erythrocytes
12.	A6-2	GalNAcα3(Fucα2)Galβ4GlcNAcβ3Galβ4Glcβ1Cer	Human erythrocytes
13.	GalNAcα3-x_2_	GalNAcα3GalNAcß3Galß4GlcNAcß3Galß4Glcß1Cer	Chicken erythrocytes
14.	A7-2	GalNAcα3(Fucα2)Galß4(Fucα3)GlcNAcß3Galß4Glcß1Cer	Human erythrocytes
15.	Neolacto-Le^x^	Galß4**GlcNAcß3Galß4**(Fucα3)**GlcNAc**ß3Galß4Glcß1Cer	Human colon cancer^c^
16.	Le^x^-7	Galß4(Fucα3)**GlcNAcß3Galß4GlcNAc**ß3Galß4Glcß1Cer	Human pancreas cancer^d^
17.	Dimeric-Le^x^	Galß4(Fucα3)**GlcNAcß3Galß4**(Fucα3)**GlcNAc**ß3Galß4Glcß1Cer	Human pancreas cancer^e^
18.	NeuGc-neolactohexa	NeuGcα3Galß4**GlcNAcß3Galß4GlcNAc**ß3Galß4Glcß1Cer	Rabbit thymus
19.	NeuAcα6-neolactohexa	Galß4GlcNAcß6(NeuAcα6Galß4GlcNAcß3)Galß4Glcß1Cer	Bovine buttermilk
20.	VIM-2	NeuAcα3Galß4**GlcNAcß3Galß4**(Fucα3)**GlcNAc**ß3Galß4Glcß1Cer	Human colon cancer
21.	NeuAcα3-Le^x^-octa	NeuAcα3Galß4(Fucα3)**GlcNAcß3Galß4GlcNAc**ß3Galß4Glcß1Cer	Human pancreas cancer
22.	NeuAcα3-dimeric-Le^x^	NeuAcα3Galß4(Fucα3)**GlcNAcß3Galß4**(Fucα3)**GlcNAc**ß3Galß4Glcß1Cer	Human pancreas cancer
23.	G9-B	Galα3(Fucα2)Galß4GlcNAcß6(NeuAcα3Galß4**GlcNAcß3**)**Galß4GlcNAc**ß3Galß4Glcß1Cer	Human erythrocytes
**II.** **Lacto binding**			
**A.** **Binding-active glycosphingolipids**			
24.	*Lactotetra*	*Galß3GlcNAcß3Galß4Glcß1Cer*	Human meconium
25.	Lacto-Le^a^	Galß3**GlcNAcß3Galß3**(Fucα4)**GlcNAc**ß3Galß4Glcß1Cer	Human small intestine
**B.** **Related non-binding glycosphingolipids**			
26.	H5-1	Fucα2Galß3GlcNAcß3Galß4Glcß1Cer	Porcine intestine
27.	Le^a^-5	Galß3(Fucα4)GlcNAcß3Galß4Glcß1Cer	Human meconium
28.	A6-1	GalNAcα3(Fucα2)Galß3GlcNAcß3Galß4Glcß1Cer	Monkey intestine
29.	Le^b^-6	Fucα2Galß3(Fucα4)GlcNAcß3Galß4Glcß1Cer	Human meconium
30.	Dimeric-Le^a^	Galß3(Fucα4)**GlcNAcß3Galß3**(Fucα4)**GlcNAc**ß3Galß4Glcß1Cer	Human small intestine
**III.** **Lactosylceramide binding**			
**A.** **Binding-active glycosphingolipids**			
31.	*GalCer*	*Galß1Cer*	Porcine intestine
32.	LacCer hydroxy	Galß4Glcß1Cer (t18∶0-h16∶0-h24∶0^f^)	Dog intestine
33.	Isoglobotri	Galα3Galß4Glcß1Cer	Dog intestine
34.	*Isoglobotetra*	*GalNAcß3Galα3Galß4Glcß1Cer*	Rat colon carcinoma
35.	Gangliotri	GalNAcß4Galß4Glcß1Cer	Guinea pig erythrocytes
36.	Gangliotetra	Galß3GalNAcß4Galß4Glcß1Cer	Mouse feces
**B.** **Related non-binding glycosphingolipids**			
37.	GlcCer	Glcß1Cer	Porcine kidney
38.	Sulfatide	SO_3_-Galß1Cer	Porcine intestine
39.	LacCer non-hydroxy	Galß4Glcß1Cer (d18∶1-16∶0 and 24∶1^f^)	Human granulocytes
**IV.** **Gal**α**3Gal**α**3Gal binding**			
**A.** **Binding-active glycosphingolipids**			
40.	Galα3-isoglobotri	Galα3Galα3Galß4Glcß1Cer	Cat intestine
41.	Galα3-B5	Galα3Galα3Galß4GlcNAcß3Galß4Glcß1Cer	Rabbit erythrocytes
**V.** **Other non-binding glycosphingolipids**			
42.	Globotri	Galα4Galß4Glcß1Cer	Human erythrocytes
43.	Globotetra	GalNAcß3Galα4Galß4Glcß1Cer	Human erythrocytes
44.	Forssman	GalNAcα3GalNAcß3Galα4Galß4Glcß1Cer	Dog intestine
45.	GalNAcα3GalNAcβ3LacCer	GalNAcα3GalNAcß3Galß4Glcß1Cer	Chicken erythrocytes
46.	GM3	NeuAcα3Galß4Glcß1Cer	Human brain
47.	GD3	NeuAcα8NeuAcα3Galß4Glcß1Cer	Bovine buttermilk
48.	GM1	Galß3GalNAcß4(NeuAcα3)Galß4Glcß1Cer	Human brain
49.	GD1a	NeuAcα3Galß3GalNAcß4(NeuAcα3)Galß4Glcß1Cer	Human brain

a) Binding is defined as a significant darkening on the autoradiogram when 1 µg of the glycosphingolipid was applied on the thin-layer plate, while binding to glycosphingolipids in.

italics require 4 µg, and non-binding denotes no binding even at 4 µg of glycosphingolipid.

b) The GlcNAcß3Galß4GlcNAc sequence, *i.e*. the common denominator for El Tor binding to lacto/neolacto based glycosphingolipids with more than four carbohydrate units is marked in bold.

c) Glycosphingolipid No. 15 was prepared from No. 20 by mild acid hydrolysis.

d) Glycosphingolipid No. 16 was prepared from No. 21 by mild acid hydrolysis.

e) Glycosphingolipid No. 17 was prepared from No. 22 by mild acid hydrolysis.

f) In the shorthand nomenclature for fatty acids and bases, the number before the colon refers to the carbon chain length and the number after the colon gives the total number of double bonds in the molecule. Fatty acids with a 2-hydroxy group are denoted by the prefix h before the abbreviation *e.g.* h16∶0. For long chain bases, d denotes dihydroxy and t trihydroxy. Thus d18∶1 designates sphingosine (1,3-dihydroxy-2-aminooctadecene) and t18∶0 phytosphingosine (1,3,4-trihydroxy-2-aminooctadecene).

When reference glycosphingolipids with terminal α3-linked Gal were examined for *V. cholerae* El Tor binding activity (Figure 7D) the bacteria bound to Galα3Galα3Galβ4Glcβ1Cer (lane 3), in addition to the Galα3Galα3Galβ4GlcNAcβ3Galβ4Glcβ1Cer from rabbit erythrocytes (lane 2). Again, no binding to the B5 pentaosylceramide (lane 1) occurred, and the B type 2 hexaosylceramide (Galα3(Fucα2)Galβ4GlcNAcβ3Galβ4Glcβ1Cer; lane 4) was also non-binding.

### Glycosphingolipid Binding by Classical *V. cholerae*

The glycosphingolipid binding of the El Tor strain and the classical strain were tested in parallel throughout the experiments described above. Although the classical strain often gave a high background, the glycosphingolipid binding pattern of the classical strain (Figure 7F) correlated well with the glycosphingolipid recognition by the El Tor strain. Thus, the classical strain also bound to neolacto- (lane 1), lacto- (lane 2), and Galα3Galα3Gal-terminated glycosphingolipids (lane 3), and also to lactosylceramide with hydroxy ceramide (not shown) and gangliotriaosylceramide (lane 5). The only exception was the El Tor binding Galβ3GlcNAcβ4Galβ3(Fucα4)GlcNAcβ3Galβ4Glcβ1Cer of human small intestine, which was not recognized by the classical strain (data not shown).

### Glycosphingolipid Recognition by a GbpA Deletion Mutant Strain

Thereafter, the role of the chitin binding protein GbpA in the binding of *V. cholerae* to glycosphingolipids was investigated by using a strain with inactivation of the *gbpA* gene. This strain displayed the same binding activities as the parental strain, *i.e.* lacto/neolacto glycosphingolipids, Galα3Galα3Gal-terminated glycosphingolipids and lactosylceramide/gangliotriaosylceramide were still recognized (exemplified in Figure 7H).

### Pretreatment of Bacterial Cells

In order to investigate the bacterial structures involved in *V. cholerae* glycosphingolipid binding the bacterial cells were subjected to pretreatments prior to the chromatogram bindng assays. Trypsin treatment and heating of the bacteria each decreased the binding to neolacto, lacto, Galα3Galα3Gal-terminated, and lactosylceramide-related glycosphingolipids (Figure S3).

## Discussion

Adhesion of microbes to target cells is considered to be an important step in the infection process, allowing an efficient delivery of toxins and other virulence factors close to the cell surface. Potential host receptors, the majority of which are glycoconjugates, have been identified for a large number of bacteria [27], [28]. However, little or no data exists regarding the potential adhesion strategies of *V. cholerae* bacterial cells. In this study, the carbohydrate binding specificity of one El Tor strain and one classical strain was examined by binding to a large number of variant glycosphingolipids. Thereby, highly specific interactions with a number of non-acid glycosphingolipids were obtained. We focused on minor complex El Tor binding compounds, which were isolated and characterized by mass spectrometry and ^1^H NMR. After comparison with the binding of the bacteria to a number of reference glycosphingolipids, three modes of El Tor glycosphingolipid recognition could be discerned.

### I. Lacto/Neolacto Binding

Most of the *V. cholerae* binding glycosphingolipids isolated and characterized in this study have lacto or neolacto core chains (Figure 5, Table 2). Lacto and neolacto glycosphingolipids are present in the human small intestinal epithelium [29]-[31], thus may contribute to *V. cholerae* colonization of the human small intestine. The *V. cholerae* binding lacto-Le^a^ glycosphingolipid (Galβ3GlcNAcβ4Galβ3(Fucα4)GlcNAβ3Galβ4Glcβ1Cer) was isolated from human small intestine, and the binding of the Galβ4GlcNAc-binding *E. cristagalli* lectin to slow-migrating glycosphingolipids of human small intestine demonstrates the presence of complex unsubstituted neolacto glycosphingolipids in the human small intestine [32]. In contrast, the *N*-linked glycans of human small intestinal glycoproteins have terminal neolacto chains which are heavily substituted with fucose [33], and thus should not be recognized by *V. cholerae*.

For high affinity binding to lacto/neolacto based glycosphingolipids a GlcNAcβ3Galβ3/4GlcNAc sequence is required (Figure 5). However, certain substitutions of the GlcNAcβ3Galβ3/4GlcNAc sequence abolished the binding of the bacteria. Thus, addition of a terminal NeuGcα3 to the terminal Gal of neolactohexaosylceramide (No. 3), yielding sialyl-neolactohexaosylceramide (No. 18), blocks the access to the binding epitope. The binding also disappears upon substitution of either of the GlcNAcs of neolactohexaosylceramide with a Fucα3 (Nos. 15 and 16). In the case of the Galβ3GlcNAcβ4Galβ3(Fucα4)GlcNAβ3Galβ4Glcβ1Cer from human small intestine (No. 25), the binding is abolished by substitution of the penultimate GlcNAc of with a Fuc in α3-position (No. 30).

The main difference between the lacto core and the neolacto core glycosphingolipids is that substitution of the innermost GlcNAc by a Fucα is tolerated for the compound with lacto core (No. 25), while it abolishes *V. cholerae* binding in the case of the glycosphingolipid with neolacto core (No. 15). This difference could be caused by conformational effects of the Fuc residues on the exposure of the binding epitope. It may also be that the lacto and neolacto sequences are recognized by two separate adhesins. The latter suggestion is somewhat supported by the fact that the classical strain fails to bind to the lacto terminated glycosphingolipid from human intestine, although it binds to the complex neolacto glycosphingolipids. Resolution of this issue must await the identification of the adhesin(s) involved in the lacto/neolacto binding.

There is a strong correlation between the severity of cholera and the blood group ABO phenotype of infected individuals, with blood group O individuals being more prone to develop severe diarrhea upon contracting *V. cholerae* infection than individuals with blood groups A, B or AB [34], [35]. However, in this study no binding of *V. cholerae* bacterial cells to glycosphingolipids with terminal blood group A, B or H determinants was obtained. A tempting speculation is that the bacteria instead might benefit from the binding to lacto and neolacto core chains, since they thereby will avoid individual variations.

Recognition of glycoconjugates with neolacto sequences is a common theme in microbial carbohydrate binding, and has also been described for *e.g. Streptococcus pneumoniae* [36], *Helicobacter pylori* [21], and relapsing fever *Borrelia* [37]. In each case the detailed structural requirements for binding differs. Interestingly, the *V. cholerae* binding GlcNAcβ3Galβ4GlcNAc sequence is also the minimum sequence recognized by *H. pylori*. However in contrast to *V. cholerae*, *H. pylori* binds to sialyl-neolactohexosylceramide, indicating a different architecture of the carbohydrate binding site of the neolacto binding adhesins.

In summary, glycosphingolipids with repetitive lacto or neolacto elements are preferentially recognized by *V. cholerae*. An increased binding is obtained by addition of a terminal β3-linked GlcNAc to neolactotetraosylceramide, indicating that the preferred binding epitope is the GlcNAcβ4Galβ4GlcNAc sequence. Since minute amounts of several of the binding-active glycosphingolipids were obtained the relative binding affinities could, unfortunately, not be accurately determined.

### II. Galα3Galα3Gal Binding

The binding to Galα3Galα3Galβ4GlcNAcβ4Galβ4Glcβ1Cer and Galα3Galα3Galβ4Glcβ1Cer is the second binding specificity of *V. cholerae*. Since the bacteria do not recognize the B5 glycosphingolipid (Galα3Galβ4GlcNAcβ4Galβ4Glcβ1Cer), the minimum binding epitope in this case is the Galα3Galα3Gal moiety. This sequence could be the product of the α1,3-galactosyltransferase or the iGb3 synthase [38], [39]. However, functional forms of these two enzymes have not been found in humans, and thus it is not likely that glycoconjugates with the Galα3Galα3Gal sequence have a role in the adhesion of *V. cholerae* to the human small intestine.

### III. Lactosylceramide Binding

The third carbohydrate binding specificity of *V. cholerae* is represented by the binding to lactosylceramide, with a concomitant binding to galactosylceramide, isoglobotriaosylceramide, gangliotriaosylceramide and gangliotetraosylceramide. The binding in the mono-, di- and triglycosylceramide region in Figure 1, Figure 7F and 7H, and Figure S3B and S3D, is thus most likely due to recognition of these compounds. The same glycosphingolipid binding pattern has previously been reported for many other bacteria, both pathogens and members of the indigenous flora [27]. While minute amounts of diglycosylceramides, most likely lactosylceramide, are present in the human intestinal epithelium [29], isoglobotriaosylceramide, gangliotri- and gangliotetraosylceramide have not been found in these cells and, thus, it is unlikely that these compounds are critical receptors for *V. cholerae*.

*V. cholerae* chitin binding protein GbpA has been proposed to mediate attachment of the bacteria to human intestine by binding to GlcNAc-containing glycoconjugates [6], [40]. We hypothesized that GbpA was involved in the binding of *V. cholerae* to the lacto/neolacto containing glycosphingolipids. However, the lacto/neolacto binding of *V. cholerae* remained after inactivation of the *gbpA* gene. Thus, the binding of *V. cholerae* to chitin and to lacto/neolacto containing glycosphingolipids represents two separate binding specificities. This is supported by the glycan array data in a recent crystalization study of GbpA, where only binding to chitin oligosaccharides was observed [7]. The GbpA deletion mutant strain also bound to the Galα3Galα3Gal-terminated compounds and the set of reference glycosphingolipid recognized by the wild type El Tor strain.

One important virulence factor of *V. cholerae* is the toxin-coregulated pilus (TCP). Expression of TCP is essential for *V. cholerae* colonization of the human small intestine [41], and it has been suggested that TCP mediates binding of the bacteria to the intestinal epithelium [42]. The culture conditions mainly used in the present study (AKI medium at 30°C) favors TCP production [43]. However, the binding-active glycosphingolipids were also recognized after culture in CFA broth at 27°C, *i.e*. under conditions when little or no TCP is expressed, indicating that the glycosphingolipid binding capacity of *V. cholerae* does not reside in the toxin-coregulated pilus.

The glycosphingolipid binding was decreased by treatment of the bacteria with trypsin and heat, indicating that bacterial cell surface proteins were involved in the interaction with carbohydrates. However, Schild *et al*. have reported that the attachment of lipopolysaccharide and capsule mutants of *V. cholerae* to the brush borders of the mucus-producing human intestinal cell line HT29-Rev MTX is reduced, demonstrating that lipopolysaccharides are important for *V. cholerae* adherence [44]. In order to investigate the role of lipopolysaccharides in *V. cholerae* glycosphingolipid binding, the bacterial cells were incubated with polyclonal antibodies to *V. cholerae* O1 antigen prior to the chromatogram binding assays. Occasionally a reduced binding to some glycosphingolipids was thereby obtained (data not shown), but the patterns were not consistent. One possible explanation for this variable reduction is that the coating of the bacterial cell surface with antibodies causes a general steric hindrance of cell surface components and adhesins that promote glycosphingolipid binding.

In this study we have for the first time defined the carbohydrate binding properties of *V. cholerae*, which is important for understanding the molecular interactions between the bacteria and its target host cells. The next step is identification, isolation and molecular cloning of the adhesins involved in the different carbohydrate binding specificities, in order to evaluate their roles in the infection process.

## Supporting Information

Figure S1 **Characterization of the El Tor** ***Vibrio cholerae*** **binding glycosphingolipid fractions TH-II, TH-III and TH-IV from rabbit thymus.** (A) MS^2^ of the [M-H^+^]^−^ ion at *m/z* 1071 (retention time 26.6 min) from LC-ESI/MS of the oligosaccharides derived from fraction TH-II. The interpretation formula shows the deduced oligosaccharide sequence. (B) MS^2^ of the [M-H^+^]^−^ ion at *m/z* 1233 (retention time 27.8 min) from LC-ESI/MS of the oligosaccharides derived from fraction TH-III. The interpretation formula shows the deduced oligosaccharide sequence. (C) MS^2^ of the [M-H^+^]^−^ ion at *m/z* 1436 (retention time 29.9 min) from LC-ESI/MS of the oligosaccharides derived from fraction TH-IV. The interpretation formula shows the deduced oligosaccharide sequence. (D) Anomeric regions of the 600 MHz proton NMR spectrum of fraction TH-IV from rabbit thymus (30^o^C). The designations F and I refer to Table 1.
(TIF)

Figure S2 **Characterization of the El Tor binding slow-migrating glycosphingolipid of dog erythrocytes.** (A) Chemical detection by anisaldehyde. (B) Autoradiogram obtained by binding of *V. cholerae* strain JBK 70. The lanes were: Lane 1, B5 pentaosylceramide (Galα3Galβ4GlcNAcβ3Galβ4Glcβ1Cer), 4 µg; Lane 2, fraction DE-I from dog erythrocytes, 4 µg; Lane 3, Galβ4GlcNAcβ3Galβ4GlcNAcβ3Galβ4Glcβ1Cer (neolactohexaglycosylceramide), 4 µg. (C) MS^2^ of the [M-2H^+^]^2−^ ion at *m/z* 880 from LC-ESI/MS of the oligosaccharide derived from the El Tor binding fraction DE-I from dog erythrocytes by hydrolysis with *Rhodococcus* endoglycoceramidase. LC-ESI/MS of the oligosaccharides obtained by hydrolysis of fraction DE-I gave a major [M-2H^+^]^2−^ ion at *m/z* 880, corresponding to a [M-H^+^]^−^ ion at *m/z* 1760, indicating a decasaccharide with three HexNAc and seven Hex. The lower mass region of the MS^2^ spectrum was weak, but had a C_3_ ion at *m/z* 544, demonstrating a terminal with two Hex and one HexNAc. In addition, C type ions at *m/z* 1436 and *m/z* 1598 were present. (D) MS^3^ of the fragment ion at *m/z* 1436 gave a C type ion at *m/z* 1233, but no further information. (E) MS^4^ of the ^0,2^A_5_ fragment ion at *m/z* 1335 gave a ^0,2^A_3_ ion at *m/z* 443, demonstrating a terminal Hex-Hex-HexNAc sequence with 4-substitution of the HexNAc. The ion at *m/z* 688 was interpreted as C_4_/Z_4α_ and C_4_/Z_4β_ ions. Thus, the MS^2^ and MS^4^ spectral features suggested a branched decasaccharide with a terminal Hex-Hex-HexNAc, *i.e.* a Hex-Hex-HexNAc-(Hex-Hex-HexNAc-)Hex-HexNAc-Hex-Hex saccharide. The interpretation formula at the top shows the deduced oligosaccharide sequence. (F) Anomeric region of the 600 MHz proton NMR spectrum of the El Tor binding glycosphingolipid of dog erythrocytes (30°C). The designation J refers to Table 1. The ^1^H NMR spectrum reveals an essentially pure compound, which is characterized by two overlapping Galα3 resonances at 4.827 ppm, two GlcNAcβ3 at 4.652 ppm, one GlcNAcβ6 at 4.395 ppm, three Galβ4 around 4.28 ppm, a fourth Galβ4 at 4.254 ppm and lastly Glcβ1 at 4.161 ppm (Table 1). These data are practically identical to previously published findings for the branched Galα3Galβ4GlcNAcβ6(Galα3Galβ4GlcNAcβ3)Galβ4GlcNAcβ3Galβ4Glcβ1Cer allowing for temperature-induced differences [24].
(TIF)

Figure S3 **Effects on glycosphingolipid binding by pretreatments of El Tor** ***Vibrio cholerae*** **bacterial cells.** Radiolabeled *V. cholerae* strain JBK 70 was incubated at 37°C for 60 min, incubated at 65°C for 60 min, or treated with trypsin at 37°C for 60 min. Thereafter the suspensions were utilized in the chromatogram binding assay. (A) Chemical detection by anisaldehyde. (B, D) Autoradiograms obtained by binding of El Tor *V. cholerae* strain JBK 70 incubated at 37°C. (C) Autoradiogram obtained by binding of El Tor *V. cholerae* strain JBK 70 treated with trypsin at 37°C. (E) Autoradiogram obtained by binding of El Tor *V. cholerae* strain JBK 70 incubated at 65°C. The lanes were: Lane 1, non-acid glycosphingolipids of rabbit thymus, 40 µg; Lane 2, lactotetraosylceramide (Galβ3GlcNAcβ3Galβ4Glcβ1Cer), 2 µg; Lane 3, Galα3Galα3Galβ4Glcβ1Cer, 2 µg; Lane 4, isoglobotriaosylceramide (Galα3Galβ4Glcβ1Cer), 4 µg. The autoradiograms displayed in B and C were from one set of experiments run in parallel, and the autoradiograms displayed in D and E were from another set of experiments run in parallel.
(TIF)
