# Supplementary figures and images for: The Repertoire of Glycosphingolipids Recognized by Vibrio cholerae
Source: PLoS One. 2013 Jan 21;8(1):e53999. doi: 10.1371/journal.pone.0053999 (PMC3549955; doi:10.1371/journal.pone.0053999)

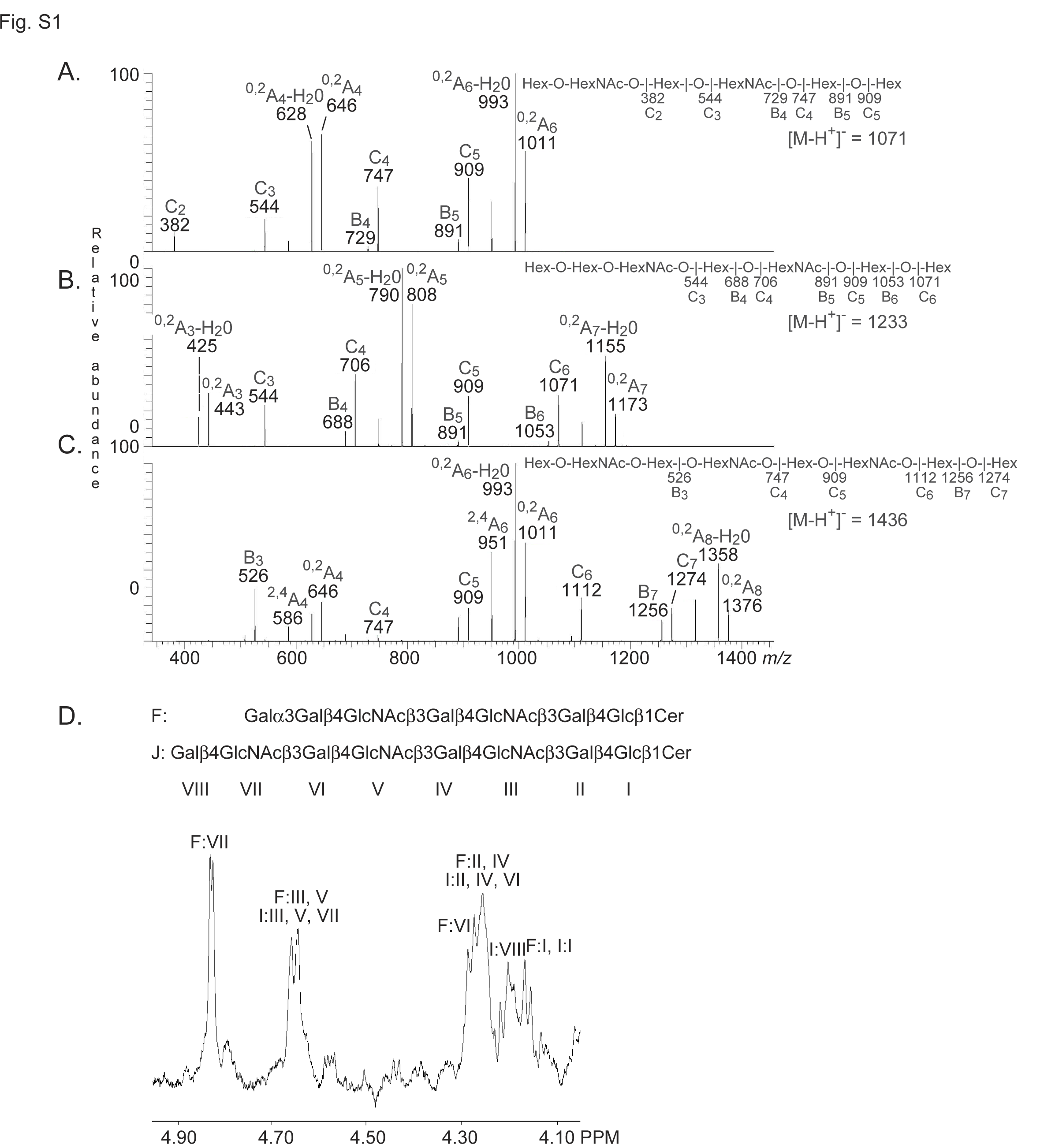

Supplement: Figure S1 — Characterization of the El Tor Vibrio cholerae binding glycosphingolipid fractions TH-II, TH-III and TH-IV from rabbit thymus. (A) MS2 of the [M-H+]− ion at m/z 1071 (retention time 26.6 min) from LC-ESI/MS of the oligosaccharides derived from fraction TH-II. The interpretation formula shows the deduced oligosaccharide sequence. (B) MS2 of the [M-H+]− ion at m/z 1233 (retention time 27.8 min) from LC-ESI/MS of the oligosaccharides derived from fraction TH-III. The interpretation formula shows the deduced oligosaccharide sequence. (C) MS2 of the [M-H+]− ion at m/z 1436 (retention time 29.9 min) from LC-ESI/MS of the oligosaccharides derived from fraction TH-IV. The interpretation formula shows the deduced oligosaccharide sequence. (D) Anomeric regions of the 600 MHz proton NMR spectrum of fraction TH-IV from rabbit thymus (30oC). The designations F and I refer to Table 1. (TIF) [file pone.0053999.s001.tif]

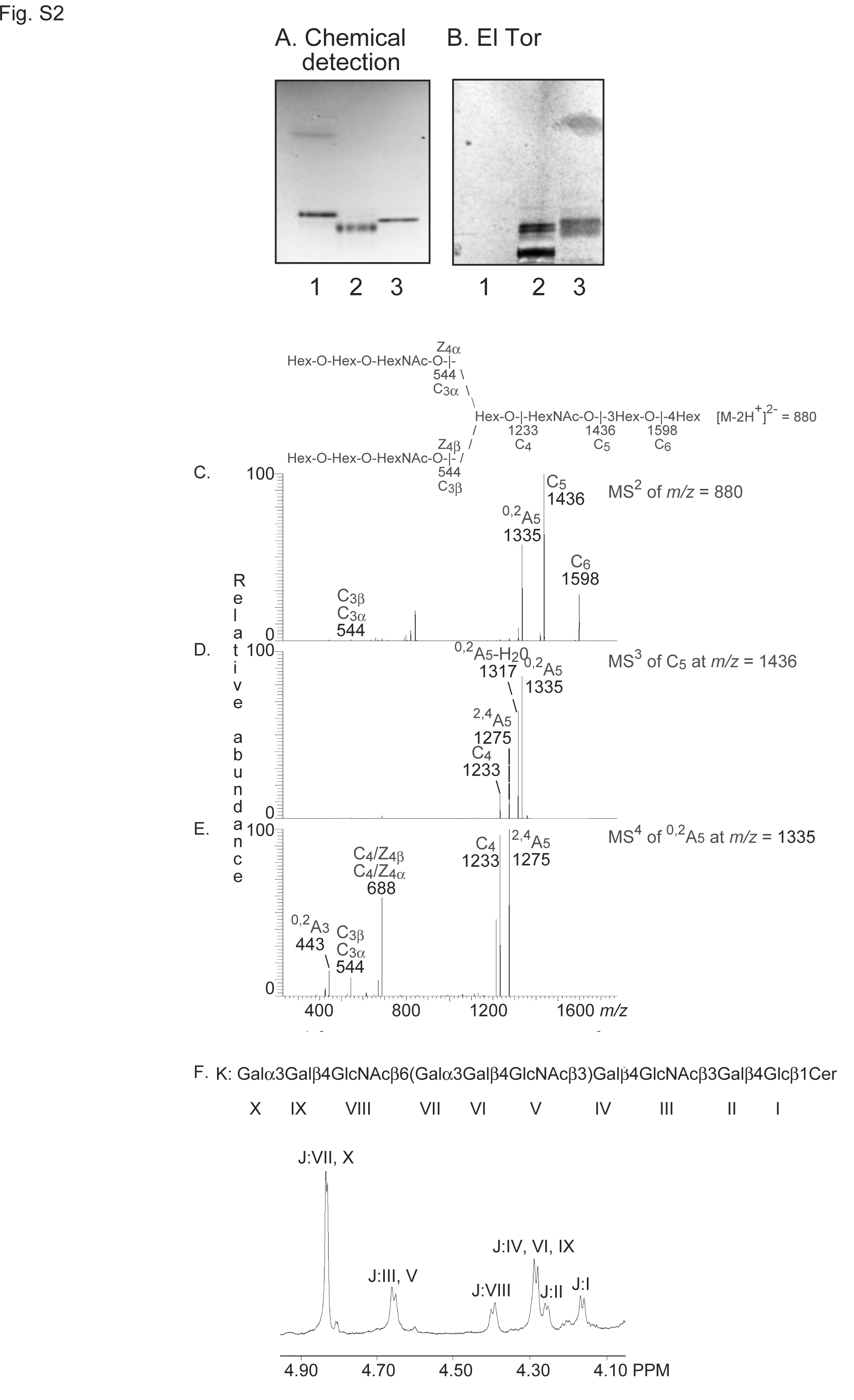

Supplement: Figure S2 — Characterization of the El Tor binding slow-migrating glycosphingolipid of dog erythrocytes. (A) Chemical detection by anisaldehyde. (B) Autoradiogram obtained by binding of V. cholerae strain JBK 70. The lanes were: Lane 1, B5 pentaosylceramide (Galα3Galβ4GlcNAcβ3Galβ4Glcβ1Cer), 4 µg; Lane 2, fraction DE-I from dog erythrocytes, 4 µg; Lane 3, Galβ4GlcNAcβ3Galβ4GlcNAcβ3Galβ4Glcβ1Cer (neolactohexaglycosylceramide), 4 µg. (C) MS2 of the [M-2H+]2− ion at m/z 880 from LC-ESI/MS of the oligosaccharide derived from the El Tor binding fraction DE-I from dog erythrocytes by hydrolysis with Rhodococcus endoglycoceramidase. LC-ESI/MS of the oligosaccharides obtained by hydrolysis of fraction DE-I gave a major [M-2H+]2− ion at m/z 880, corresponding to a [M-H+]− ion at m/z 1760, indicating a decasaccharide with three HexNAc and seven Hex. The lower mass region of the MS2 spectrum was weak, but had a C3 ion at m/z 544, demonstrating a terminal with two Hex and one HexNAc. In addition, C type ions at m/z 1436 and m/z 1598 were present. (D) MS3 of the fragment ion at m/z 1436 gave a C type ion at m/z 1233, but no further information. (E) MS4 of the 0,2A5 fragment ion at m/z 1335 gave a 0,2A3 ion at m/z 443, demonstrating a terminal Hex-Hex-HexNAc sequence with 4-substitution of the HexNAc. The ion at m/z 688 was interpreted as C4/Z4α and C4/Z4β ions. Thus, the MS2 and MS4 spectral features suggested a branched decasaccharide with a terminal Hex-Hex-HexNAc, i.e. a Hex-Hex-HexNAc-(Hex-Hex-HexNAc-)Hex-HexNAc-Hex-Hex saccharide. The interpretation formula at the top shows the deduced oligosaccharide sequence. (F) Anomeric region of the 600 MHz proton NMR spectrum of the El Tor binding glycosphingolipid of dog erythrocytes (30°C). The designation J refers to Table 1. The 1H NMR spectrum reveals an essentially pure compound, which is characterized by two overlapping Galα3 resonances at 4.827 ppm, two GlcNAcβ3 at 4.652 ppm, one GlcNAcβ6 at 4.395 ppm, three Galβ4 around 4.28 ppm, a fourt [file pone.0053999.s002.tif]
